# Assessment by Surface Electromyography in International Football Players with Cerebral Palsy—A Pilot Study

**DOI:** 10.3390/jfmk10020125

**Published:** 2025-04-10

**Authors:** Alejandro Caña-Pino, María Dolores Apolo-Arenas, Iván Peña-González

**Affiliations:** 1 Surgical Medical-Therapy Department, Medicine Faculty and Health Sciences, University of Extremadura, 06006 Badajoz, Spain; alejandrocp@unex.es; 2 Research Group PhysioH (Fisioterapia e Hipoterapia), University of Extremadura, 06006 Badajoz, Spain; 3 Spanish Federation of Sports for People with CP and ABI (FEDPC), 28008 Madrid, Spain; ipena@umh.es; 4 Sports Research Centre, Department of Sports Sciences, Miguel Hernández University of Elche, 03202 Elche, Spain

**Keywords:** Cerebral Palsy Football, para-sport, electromyography, asymmetry, sport classification

## Abstract

**Background:** In para-sports, like Cerebral Palsy (CP) Football, athletes must meet a minimum impairment level to ensure fair competition. The classification process traditionally relies on subjective tools like the modified Ashworth Scale, but there is a need for more objective methods. Surface electromyography (EMG) offers quantifiable data on muscle activation, which could enhance the accuracy and fairness of classification in this sport. **Objective:** The aim of this study is to analyze muscle activation patterns in international CP football players compared to healthy controls, using surface electromyography (EMG). **Methods:** A cross-sectional, observational case–control study (following STROBE guidelines) was carried out. The final sample consisted of 40 subjects (20 subjects with CP from the Spanish National Team and 20 semi-professional able-bodied football players). The muscle activation of the soleus, adductor magnus, and biceps femoris was evaluated at baseline and in maximum isometric contraction in both dominant/unaffected and non-dominant/affected lower limbs. **Results:** The main result of this study was that the affected lower limbs of the experimental group showed higher muscle activation at baseline compared to those of the control group (*p* < 0.001). On the other hand, when a maximum isometric contraction was requested, muscle activation was greater in the control group in both lower limbs. There was greater asymmetry between both muscle groups in the experimental group. **Conclusions:** Surface electromyography could be a useful tool to be used in the assessment of muscle activity in subjects with CP with an applicability in para-sport, making it possible to obtain differences between both hemispheres when there is upper motor neuron involvement.

## 1. Introduction

The classification process in para-sports, like Cerebral Palsy (CP) Football, ensures fair competition by verifying that players meet the eligibility criteria and determining the severity of their impairments [1]. The International Paralympic Committee’s (IPC) 2015 Athlete Classification Code requires all para-sports to adopt evidence-based, sport-specific classification systems focused on the relationship between the impairment and the key performance determinants [2].

CP football, a para-sport practiced in 77 countries, is designed for athletes with CP or acquired brain injury. The game follows the International Football Association Board’s rules, with specific modifications, as follows: seven players per team, two 30 min halves, smaller pitch and goals, no offside rule, and throw-ins executed by rolling the ball. To be eligible to participate in CP football, athletes must meet a minimum impairment criterion of hypertonia, athetosis, or ataxia (HAA), which are three of the eight eligible impairments in Paralympic sport [3].

The CP population is characterized by impaired motor control, which is a consequence of most central nervous system (CNS) movement disorders, such as CP, stroke, spinal cord injury, and multiple sclerosis (MS) [4,5,6]. A common physical examination includes an assessment of passive muscle stretching endurance, isometric, and isotonic tests [7]. These tests are used to judge the degree and nature of muscle hyper-resistance in order to determine the etiology at the level of muscle tissue and/or motor control, in addition to inferring the consequences for overall motor performance in functional tasks. This is considered important for a meaningful description of the patient’s clinical condition and is essential to inform decisions about treatment options [4,5,6].

The impacts on sports performance caused by impaired motor control due to CP include specific alterations depending on the type of impairment [8]. In spastic hypertonia, there is an increase in muscle tone, hyperreflexia, clonus, muscle contractures, and a limited range of motion. Ataxia, caused by cerebellar dysfunction, is characterized by poor coordination, uncoordinated movements with irregular amplitude and rhythm, and difficulties in maintaining balance, resulting in instability in muscular movements [8]. On the other hand, athetosis, associated with extrapyramidal lesions, manifests as slow, writhing involuntary movements, primarily in the distal extremities, with fluctuations in muscle tone that hinder precision and postural stabilization [8]. All of these limitations could restrict the ability to perform specific game actions, such as running, dribbling, passing, or shooting the ball, directly impacting performance in CP football [1].

Among the tests used in the medical assessment process for classification in CP football, validated methods are employed to evaluate spastic hypertonia, ataxia, and athetosis. Spasticity or hypertonia is assessed using the modified Ashworth Scale (MAS), which measures muscle tone during passive movement in a supine position, with resistance graded from 0 (no increase in muscle tone) to 4 (complete rigidity), as described by Bohannon and Smith [9]. Ataxia is evaluated using the Scale for the Assessment and Rating of Ataxia (SARA), which includes specific tests such as gait, stance, finger chase, and heel-to-shin slide, based on the work of Schmitz-Hübsch et al. [10]. Athetosis and dystonia are assessed using a modified version of the Dyskinesia Impairment Scale (DIS), developed by Monbaliu et al. [11], which examines the amplitude and duration of abnormal movements across different body regions. These tests, along with additional methods, such as the Berg Balance Scale, ensure a standardized and accurate classification process for athletes in CP football. However, the evaluation of spastic hypertonia, ataxia, and athetosis in CP football players using the aforementioned tests relies primarily on the subjective interpretation of the observer, which can present a challenge in maintaining high intra- and inter-observer reliability. These tests, while standardized, depend on the evaluator’s perception and clinical judgment, such as assessing muscle resistance in the MAS or identifying movement abnormalities in the DIS. This inherent subjectivity underscores the need for experienced evaluators to ensure consistency and accuracy.

Surface electromyography (sEMG) is a non-invasive technique that measures the electrical activity of muscles using electrodes placed on the skin, allowing for the analysis of muscle activation patterns both at rest and during movement. sEMG evaluates the intensity and synchronization of muscle contractions, enabling the detection of abnormalities such as hypertonia, the involuntary movements characteristic of athetosis, and the inefficient coordination associated with ataxia. Assessing muscle activity in athletes with CP through sEMG holds significant potential in the context of sports classification, as it provides objective and quantifiable data that complement traditional subjective tools. Specifically, the use of sEMG in CP football players could enhance the classification process by refining the determination of minimal impairment criteria—ensuring eligibility based on the minimum impact required for participation—and by aiding in the assignment of sport classes. Additionally, sEMG offers practical applications for optimizing athletic performance and preventing injuries, all grounded in objective data and scientific evidence.

Given the above factors, the aim of this pilot study was to analyze muscle activation patterns in international CP football players compared to able-bodied footballers using sEMG. This study aims to identify differences in baseline and maximal isometric contraction between CP footballers, characterized by a minimum impairment in spastic hypertonia, athetosis, and ataxia, compared to semi-professional footballers without disability. The findings seek to contribute to the development of an objective, direct, reliable, and applicable evaluation method that complements traditional subjective tools, thereby enhancing the accuracy of the sports classification system.

## 2. Materials and Methods

This is a cross-sectional case–control study conducted in accordance with the Strengthening the Reporting of Observational Studies in Epidemiology (STROBE) statement [12] and the Declaration of Helsinki modified by the 64th General Assembly of the World Medical Association (Fortaleza, Brazil, 2013). This study was approved by the Ethical Research Committee of the University of Extremadura, Spain (project code 81//2022, approval date: 16 June 2022), and was registered at clinicaltrials.gov with registration number NCT06151873.

### 2.1. Sample Size Calculation

A convenience sample of 20 participants per group was considered in this study. This sample size was considered to be sufficient to detect an effect size of δ = 0.50, given a 2-sided level, 5% paired sampled *t*-test and a statistical power of 80%. The sample size was calculated using Jamovi 1.6 computer software, the Jamovi project (2020) [13].

### 2.2. Settings and Participants

The eligible potential sample was composed of 45 participants (Control Group [CG]: 25 semi-professional football players recruited from group II of the 1st division of Extremadura football; Experimental Group [EG]: 20 international football players with CP/ABD from the Spanish National Team, world ranked 9th of 84 national teams. The inclusion criteria for the EG were as follows: (a) having a diagnosis of CP or acquired brain injury; (b) belonging to the senior team of the Spanish National CP Football Team; and (c) being federated in the Spanish Sports Federation of People with Cerebral Palsy. The inclusion criteria for the CG were as follows: (a) to present a federative license in the sport of football; and (b) belonging to a senior team in group II of the 1st division of Extremadura football. The exclusion criteria for both groups were as follows: (a) having any injury of musculoskeletal origin at the time of the assessment or in the six months prior to the assessment. The participants were informed about the nature of this study, and written informed consent was obtained from all participants prior to participation.

**Figure 1:**
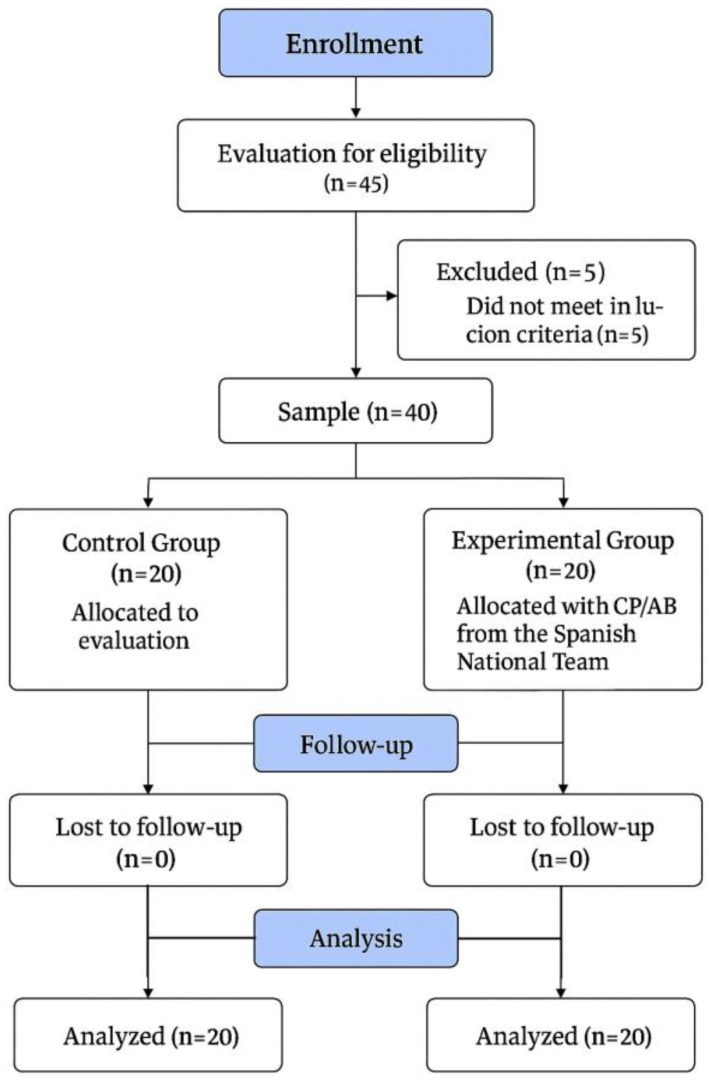
Flowchart of this study.

After the application of the exclusion criteria, 40 participants were finally included in this study (Figure 1). The EG was finally composed of 20 male international CP football players (age: 25.9 ± 6.57 years, body mass: 68.8 ± 8.13 kg, body height: 1.74 ± 0.07 m, BMI: 22.5 ± 2.39 kg·m^2^, training frequency: 3.85 ± 0.22 days per week). This group was also composed of 5 players with bilateral spastic hypertonia, 3 players with athetosis/ataxia, and 12 players with unilateral spasticity. On the other hand, the CG was finally composed of twenty male football players (age: 22.6 ± 4.07 years, body mass: 71.5 ± 9.34 kg, body height: 1.76 ± 0.07 m, BMI: 23.3 ± 2.52 kg·m^2^, training frequency: 2.9 ± 0.31 days per week).

### 2.3. Material

The mDurance^®^ system (mDurance Solutions SL, Granada, Spain) is a portable and low-cost sEMG system that consists of three parts. First, there is a Shimmer3 EMG unit (Realtime Technologies Ltd., Dublin, Ireland), which is a bipolar sEMG sensor for the acquisition of superficial muscle activity. Each Shimmer sensor is composed of two sEMG channels, with a sampling rate of 1024 Hz. Shimmer applies a bandwidth 8.4 kHz (built-in filter of 20–450 Hz), the EMG signal resolution is 24 bits with an overall amplification of 100–10,000 V/V, the EMG signal resolution is 16 bits, and the inter-sensor latency is <500 us. The electrodes used were pre-gelled Ag/AgCl with a diameter of 10 mm and an interelectrode distance of 20 mm. Second, the mDurance 2.20.0 (Android) mobile application is responsible for receiving data from the Shimmer unit and sending it to a cloud service. Third, the mDurance cloud service is where the sEMG signals are stored, filtered, and analyzed, generating the reports. The mDurance^®^ system has been previously validated with excellent relative validity and fair to good absolute validity, with a systematic bias of 1.62 ± 8% random error for the mean [14]. In addition, the recommendations of the International Society of Electrophysiology and Kinesiology have been followed [15].

### 2.4. Procedures

One experienced independent physiotherapist who assessed the suitability of each participant, based on the inclusion and exclusion criteria, examined the participants at the baseline. These researchers were not involved in the measurements. The assessment protocol was as follows: the participants, after a short warm-up of 3 min based on joint mobility and a short rest, proceeded to have the electromyographic activity of the following muscles assessed bilaterally: soleus, adductor magnus, and biceps femoris. These muscles have been selected because they are the muscles valued in the CP football classification system, due to their clear impact on the game (IFCPF, 2024) [3]. The electrode placement protocol for recording was described by Molina et al. [14]. The muscle activity was measured at the baseline and at maximum isometric contraction (MIC) of each muscle pair. The duration of each recording was 5 s. All assessments were carried out on both the non-dominant or affected leg (the leg with the greatest impact of the disability and the lesser ability to play football) and on the musculature of the dominant or non-affected (which could be “less affected”) leg (the leg with no or lower impact of the disability and with a greater ability to play football). All data were recorded with the system and transferred and restored to a portable computer via a USB cable for further analysis.

**Figure 2:**
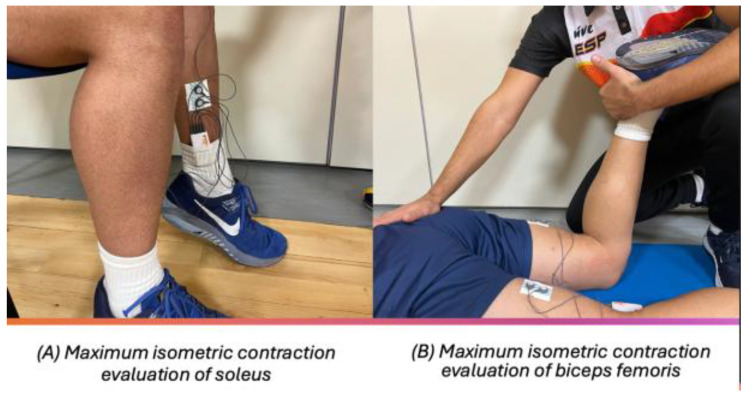
Subject position for evaluation.

The assessment of the soleus and adductor magnus musculature was carried out as follows (Figure 2): The participants were seated on a chair or bench, with feet flat on the floor at a 90° angle at both hips and knees. Their back was straight, and they had relaxed arms resting on the thighs. On the other hand, for the assessment of the biceps femoris muscle, the following took place: The participants were in a prone position on a stretcher. The lower limb to be assessed was placed with lower knee flexion of 90°, with the opposite lower limb in extension. The foot of the non-evaluated lower limb was slightly off the stretcher. The arms were relaxed along the trunk (Figure 2). The order of the tests was as follows, without randomization in basal and isometric situation: soleus, adductor magnus, and biceps femoris.

### 2.5. Measures

Several descriptive measurements were collected for sample characterization, including age, body mass, body height, Body Mass Index (BMI), training frequency (measured in days per week), and type of affectation (bilateral spasticity; athetosis/ataxia; and unilateral spasticity). The root mean square (RMS) of the EMG signals was calculated based on the Fourier transform. This indicator is a smoothing of the EMG signal to facilitate its interpretation. It is also the most widely used in the literature [14,16,17], and it was expressed in microvolts (μv).

### 2.6. Statistical Analysis

The statistical analyses were performed using the Jamovi project (2020) [13]. The significance level was set at *p* < 0.05. A descriptive analysis was performed for each of the variables (age, body mass, body height, Body Mass Index (BMI), and training frequency). The sample was tested for normality using the Shapiro–Wilk test. It did not follow a normal distribution and, therefore, non-parametric tests were performed. The descriptive data included means and standard deviations (SDs). The variables of both intervention groups at the baseline were compared with the independent samples *t*-test. To compare both within-group and between-group difference, we applied non-parametric models: the Wilcoxon test and Mann–Whitney test, respectively. The within-group comparison involved analyzing the sEMG data between limbs (dominant/unaffected vs. non-dominant/affected). The analysis was performed for the three muscles studied (soleus, adductor magnus, and biceps femoris) and across two states of muscle activation: baseline and maximum isometric contraction (MIC). The pairwise effect size (ES) for both intra- and intergroup comparisons was determined using Hedges’ g index [18]. Unlike Cohen’s d index [19], Hedges’ g index adjusts for small sample sizes (n < 20), providing a less biased estimation of the ES. Hedges’s g index was interpreted as follows: a result greater than 0.8 indicates a large effect, 0.5–0.8 a moderate effect, 0.2–0.5 a small effect, and less than 0.2 a trivial effect.

## 3. Results

There were twenty male subjects in the EG (age: 25.9 ± 6.57 years, body mass: 68.8 ± 8.13 kg, body height: 1.74 ± 0.07 m, BMI: 22.5 ± 2.39 kg·m^2^, training frequency: 3.85 ± 0.22 days per week). In addition, there were twenty male subjects in the CG (age: 22.6 ± 4.07 years, body mass: 71.5 ± 9.34 kg, body height: 1.76 ± 0.07 m, BMI: 23.3 ± 2.52 kg·m^2^, training frequency: 2.9 ± 0.31 days per week). The independent samples *t*-test did not show initial differences between the groups for age (years) (t = −1.91, *p* = 0.06), body height (meters) (t = 0.63, *p* = 0.53), body weight (kg) (t = 0.96, *p* = 0.35), BMI (kg·m^2^) (t = 1.01, *p* = 0.32), and training frequency (days per week) (t = 0.97, *p* = 0.71).

The CG showed similar muscular activation between the dominant and non-dominant lower limbs, as follows: soleus baseline (μv) (t = −1.11 to 0.69, *p* > 0.05); soleus MIC (μv) (t = −13.12 to 23.98, *p* > 0.05); adductor magnus baseline (μv) (t = −1.98 to 0.13, *p* > 0.05); adductor magnus MIC (μv) (t = −29.94 to 11.20, *p* > 0.05); biceps femoris baseline (μv) (t = −0.89 to 1.07, *p* > 0.05); and biceps femoris MIC (μv) (t = −28.86 to 32.06, *p* > 0.05) (Table 1). Therefore, the muscle activation between the lower limbs in the CG was similar both in the basal state and in MIC.

The EG showed significant differences for the muscular activation between the unaffected (dominant) and affected (non-dominant) lower limbs in all muscle groups studied (soleus, adductor magnus, and biceps femoris) both for the basal state and MIC (*p* < 0.001). In the basal state, greater activation was obtained in the affected lower limb, while, for MIC, greater activation was obtained for the unaffected lower limb (Table 1).

In the basal state, a greater activation of the adductor magnus muscle of the affected lower limb was obtained. However, when the maximum isometric contraction was evaluated, greater activation of the biceps femoris of the unaffected lower limb was obtained. The soleus in the baseline presented significant differences between the groups for the dominant/unaffected lower limb, while the adductor magnus in the baseline presented significant differences between the groups for the non-dominant/affected lower limb.

On the other hand, in the intergroup analysis, statistically significant differences were obtained in the activation of the soleus in the basal state (*p* < 0.001), where the activation was greater in the control group. (Table 2). However, when the affected lower limb of the experimental group was compared with the non-dominant lower limb of the control group, statistically significant differences were obtained for the adductor magnus musculature (*p* = 0.045) (Table 2).

The players for the EG presented higher asymmetries than those of the CG for the soleus baseline (μv) (EG: 2.68 ± 4.51, CG: 0.37 ± 1.67, t = 2.05, *p* = 0.01); soleus MIC (μv) (EG: 53.04 ± 57.32, CG: 4.74 ± 19.21, t = −3.17, *p* = 0.004); adductor magnus baseline (μv) (EG: 3.34 ± 4.67, CG: 0.46 ± 0.82, t = 2.67, *p* = 0.001); adductor magnus MIC (μv) (EG: 85.66 ± 78.75, CG: 7.52 ± 23.2, t = −4.65, *p* = 0.001); biceps femoris baseline (μv) (EG: 3.23 ± 5.54, CG: 0.65 ± 4.29, t = 2.33, *p* = 0.02); and biceps femoris MIC (μv) (EG: 67.72 ± 69.85, 6.38 ± 64.77, t = −2.46, *p* = 0.003).

## 4. Discussion

The primary aim of this study was to evaluate sEMG as an assessment tool for health professionals in clinical settings and sports practitioners working with individuals with CP, particularly in the context of para-sport, like CP football. The findings demonstrate that sEMG can effectively quantify muscle activation patterns, revealing significant differences between individuals with neurological impairments and able-bodied controls.

A key result was that the affected lower limb of the EG exhibited significantly higher muscle activation at the baseline compared to that of the CG. Conversely, during maximum isometric MIC, the CG displayed greater muscle activation in both lower limbs. This contrast highlights a notable asymmetry in muscle activation between the affected and unaffected limbs in the EG, a finding consistent with the expected motor impairment profile of unilateral neurological disorders such as CP [20]. The EG showed a greater asymmetry in muscle activation between the lower limbs compared to the CG, which is consistent with the previous literature, as it is a common pattern for people with unilateral neurological disorders, such as those found in some cases of CP [7,21]. These results suggest that sEMG could serve as a valuable tool for assessing muscle function in athletes with disabilities, both at rest and during maximal effort. For instance, Gagnat et al. [22] have similarly endorsed sEMG as a clinical tool for evaluating muscle activity during functional tasks like walking in children with CP, reinforcing its utility in dynamic contexts.

The observed asymmetry in the EG—showing higher baseline activation in the affected limb and reduced activation during MIC compared to the unaffected limb—aligns with the pathophysiology of spasticity. Campanella et al. [23] described spasticity as a positive symptom of upper motor neuron impairment, characterized by increased muscle tone at rest (e.g., spastic dystonia) and following passive stretching. This is corroborated by studies noting elevated resting muscle activity in spastic muscles, even in neutral or shortened positions, despite attempts at relaxation [5]. Similarly, Wang et al. [24] reported greater baseline activation in the tibialis anterior and calf muscles of children with CP compared to controls, alongside reduced activity and lower motor unit recruitment during active contraction. These patterns reflect the impaired neuromuscular control typical of CP, where spastic muscles exhibit hypertonia at rest but fail to generate proportional force during voluntary effort.

Further insights emerge from dynamic movement analyses. Gagnat et al. [22] found that children with CP exhibit increased soleus activation during the weight acceptance phase of gait compared to their typically developing peers, yet lower activation across other gait phases. They also noted heightened hamstring activity in the CP group, suggesting that spasticity or spastic dystonia contributes to increased muscle tone during movement, particularly as speed varies. Marinelli [25] supports this, emphasizing that spasticity and dystonia are velocity-dependent phenomena. However, some studies [26,27] have found no significant differences between paretic and non-paretic limbs in individuals with CP, though they consistently observed greater activation in hypertonic muscles compared to non-hypertonic ones in healthy controls [23]. This suggests that spastic muscles may disproportionately influence motor tasks by activating synergists in an uncoordinated manner, potentially disrupting the balanced recruitment of other muscle groups.

Clinically, these findings underscore sEMG’s potential as a quantitative tool to differentiate spastic musculature from non-spastic musculature. With further validation, sEMG could establish standardized cut-off points for classifying spasticity severity, enhancing diagnostic precision in clinical and sports settings. In the context of para-sports, such as CP football, sEMG could objectively assess muscle imbalances and activation asymmetries, informing training strategies to optimize performance and reduce injury risk. For example, identifying hypertonic muscles that limit the range of motion or coordination could guide targeted interventions. In this sense, sEMG is not intended to replace traditional assessments, but to complement them. Its use provides a more objective and quantifiable analysis of muscle activation, which helps to improve the accuracy of the sports classification system.

Despite these promising implications, this study’s small sample size (n = 40) limits its generalizability. Another limitation is that the placement of sEMG electrodes could be affected by individual variations in muscle anatomy. On the other hand, the quality of the electromyographic signal could be influenced by factors such as sweating, electrode movement, and environmental electrical activity, which could introduce noise in the data. Although differences in muscle activation between the groups were identified, we did not evaluate how these differences affected the athletes’ sporting or functional performance in a real competition. Future research should involve larger cohorts and explore additional variables, such as muscle fatigue, differences across neurological impairment types (e.g., spastic diplegia vs. hemiplegia), different levels of disability, and variations within international sport classification categories. Moreover, while this study focused on static conditions (baseline and MIC), extending sEMG analysis to dynamic tasks like running or kicking—key actions in CP football—could further validate its applicability in sports performance contexts. It could also be applied to a wider variety of adapted sports to analyze its applicability and objectivity. Finally, this study focused on muscle activation in specific conditions, but an analysis of activation patterns in dynamic or functional tasks could provide additional information relevant to sports classification.

Surface electromyography offers practical applications that directly support this study’s aim of enhancing the accuracy and objectivity of sports classification in CP football. By providing quantifiable data on muscle activation patterns, sEMG can refine the determination of minimal impairment criteria and improve sport class assignments by detecting asymmetries between affected and unaffected limbs, addressing the subjectivity of traditional tools like the modified Ashworth Scale. Beyond classification, sEMG enables tailored training programs to optimize athletic performance by targeting neuromuscular imbalances, supports injury prevention through the identification of hypertonic muscles, and serves as a clinical tool for monitoring motor impairments, thus contributing to a more evidence-based, fair, and precise classification system for CP football players.

## 5. Conclusions

Surface electromyography has emerged as a promising tool for assessing muscle activation in individuals with CP, with clear applications in para-sports. By detecting differences in muscle activity between hemispheres in cases of upper motor neuron involvement, sEMG offers an objective complement to traditional, subjective classification methods, potentially enhancing fairness and accuracy in CP football classification.

## Figures and Tables

**Table 1:** Intragroup analysis. Muscle activity RMS.

	CG (n = 20) EG (n = 20)	Dominant/Unaffected Lower Limb (Mean ± SD)	Non-Dominant/ Affected Lower Limb (Mean ± SD)	Asymmetry (Mean ± SD)	t	*p* ^τ^	ES (95% CI)
Soleus Baseline (μv)	CG	4.05 ± 1.42	4.42 ± 2.38	0.37 ± 1.67	−0.76	0.927	−0.17 (−0.61, 0.27)
	EG	2.85 ±1.19	5.52 ± 5.08	2.68 ± 4.51	−2.65	<0.001	−0.59 (−1.06, −0.11)
Soleus MIC (μv)	CG	112.86 ± 47.72	108.12 ± 40.34	4.74 ± 19.21	0.57	0.571	0.13 (−0.31, 0.57)
	EG	137.26 ± 79.69	84.22 ± 36.91	53.04 ± 57.32	4.14	<0.001	0.93 (0.39, 1.44)
Adductor magnus Baseline (μv)	CG	3.85 ± 1.25	4.31 ± 1.65	0.46 ± 0.82	−1.75	0.112	−0.39 (−0.84, 0.07)
	EG	4.21 ± 2.69	7.55 ± 7.14	3.34 ± 4.67	−3.20	<0.001	−0.72 (−1.20, −0.21)
Adductor Magnus MIC (μv)	CG	143.26 ± 81.16	150.78 ± 72.80	7.52 ± 23.2	−0.79	0.388	−0.18 (−0.62, 0.27)
	EG	223.76 ± 151.68	138.10 ± 93.17	85.66 ± 78.75	4.86	<0.001	1.09 (0.52, 1.64)
Biceps Femoris Baseline (μv)	CG	5.28 ± 4.48	4.63 ± 3.20	0.65 ± 4.29	0.58	0.852	0.13 (−0.31, 0.57)
	EG	3.70 ± 1.96	6.93 ± 6.63	3.23 ± 5.54	−2.61	<0.001	−0.58 (−1.05, −0.10)
Biceps Femoris MIC (μv)	CG	201.94 ± 92.93	195.56 ± 69.62	6.38 ± 64.77	0.33	0.869	0.07 (−0.37, 0.51)
	EG	238.44 ± 139.46	170.72 ± 125.22	67.72 ± 69.85	4.34	<0.001	0.97 (0.43, 1.50)

^τ^: Wilcoxon test; SD: standard deviation; CG: control group (healthy subjects); EG: experimental group (patients with CP/ABD from the Spanish National Team); ES: effect size; CI: confidence interval; μv: microvolt.

**Table 2:** Intergroup analysis. Muscle activity RMS.

	Domminant/Unaffected				Non-Dominant/Affected			
	CG	EG	t (*p* ^τ^)	ES (95% CI)	CG	EG	t (*p* ^τ^)	ES (95% CI)
Soleus Baseline (μv)	4.05 ± 1.42	2.85 ±1.19	2.90 (<0.001)	0.92 (0.26; 1.56)	4.42 ± 2.38	5.52 ± 5.08	−0.88 (0.839)	−0.28 (−0.90; 0.35)
Soleus MIC (μv)	112.86 ± 47.72	137.26 ± 79.69	−1.17 (0.529)	−0.37 (−0.99; 0.26)	108.12 ± 40.34	84.22 ± 36.91	1.95 (0.063)	0.62 (−0.02; 1.25)
Adductor magnus Baseline (μv)	3.85 ± 1.25	4.21 ± 2.69	−0.55 (0.617)	−0.18 (−0.80; 0.45)	4.31 ± 1.65	7.55 ± 7.14	−1.98 (0.045)	−0.63 (−1.26; 0.01)
Adductor Magnus MIC (μv)	143.26 ± 81.16	223.76 ± 151.68	−2.09 (0.063)	−0.66 (−1.30; −0.02)	150.78 ± 72.80	138.10 ± 93.17	0.48 (0.369)	0.15 (−0.47; 0.77)
Biceps Femoris Baseline (μv)	5.28 ± 4.48	3.70 ± 1.96	1.45 (0.140)	0.46 (−0.17; 1.08)	4.63 ± 3.20	6.93 ± 6.63	1.40 (0.310)	−0.44 (−1.07; 0.19)
Biceps Femoris MIC (μv)	201.94 ± 92.93	238.44 ± 139.46	−0.97 (0.394)	−0.31 (−0.93; 0.32)	195.56 ± 69.62	170.72 ± 125.22	0.78 (0.120)	0.25 (−0.38; 0.87)

^τ^: Mann–Whitney test; CG: control group (healthy subjects); EG: experimental group (patients with CP/ABD from the Spanish National Team); ES: effect size; CI: confidence interval; μv: microvolt.

## Data Availability

Data are contained within the article.
